# Dissociative Trance Led to a Catastrophe: A Case Report

**DOI:** 10.7759/cureus.76458

**Published:** 2024-12-27

**Authors:** Ahmed Z Salama, Mohammad R Dabash, Abdullah Elayan, Shafee Saadeh, Ibrahim Ikhmayyes

**Affiliations:** 1 School of Medicine, Al-Quds University, Jerusalem, PSE; 2 Psychiatry, Dr. Kamal Psychiatric Hospital, Bethlehem, PSE

**Keywords:** amnesia, cognitive behavioral therapy, dissociative trance, emotional stress, mental health

## Abstract

Dissociation is a cognitive process that disrupts consciousness, identity, or memory. It is frequently used as a form of defense in response to significant stress or trauma. In serious situations, it might show as a dissociative disorder, which extremely impairs psychological functioning. Dissociative trance, a type of other specified dissociative disorder (OSDD), is characterized by an abrupt loss of awareness, leading to unresponsiveness and involuntary actions. Despite being conscious, the individual may feel separated from their body, as if it were under the control of an external force.

This case report represents a 22-year-old patient with no prior criminal history who experienced episodes of decreased consciousness, altered identity, and involuntary movements. These episodes were perceived as being controlled by an external force and culminated in a tragic event leading the patient to kill her children. After ruling out other potential causes like psychotic disorders, organic factors, and substance abuse, a diagnosis of dissociative trance disorder was considered. Treatment with cognitive behavioral therapy, antidepressants, antipsychotics, and benzodiazepines resulted in improvement of the patient's symptoms.

## Introduction

Dissociation is a cognitive process that interrupts the regular integration of consciousness, identity, memory, perception, emotion, behavior, motor control, and bodily representation [1]. It is a common defense strategy for coping with extreme stress or trauma. Mild dissociation is like daydreaming or driving along a familiar road and then realizing you can't recall the last few kilometers [2]. In certain circumstances, dissociation manifests as a more serious condition or as persistent, chronic behavior that disrupts many aspects of psychological functioning, known as dissociative disorder.

Dissociative disorders are prevalent mental health illnesses, affecting around 10% of individuals in clinical settings. The most severe type, dissociative identity disorder (DID), accounts for around 5% of all cases. These disorders are commonly associated with severe childhood trauma, such as sexual abuse (57.1%-90.2%), emotional abuse (57.1%), physical abuse (62.9%-82.4%), and neglect (62.9%). Individuals with dissociative disorders frequently present in crises, requiring immediate intervention. Certain populations, such as exotic dancers, sex workers, and vulnerabilities, are considered the most vulnerable groups in society to dissociative disorders. Unfortunately, specific pharmaceutical therapies for dissociative disorders are limited due to their complexity and frequent coexistence with other mental conditions. Effective treatment usually involves a combination of therapeutic modalities, including psychotherapy and, in some situations, medication to manage comorbid problems [3].

These disorders appear in many forms, including dissociative identity disorder (DID), which is identified by the presence of two or more separate personality states, a sensation of possession, and recurrent amnesia. Dissociative amnesia (DA) refers to the inability to remember personal information; this amnesia can be generalized when an individual's identity or life history is lost, localized when an event or time period is absent, or selective when only a portion of an event is missing. Depersonalization/derealization disorder (DPDRD) is marked by recurrent or persistent depersonalization, which involves feeling detached from one's self, body, or mind. It can occur alone or in conjunction with derealization, which involves feeling detached from one's surroundings [1].

Other specified dissociative disorders (OSDD) occur when symptoms of a dissociative disorder predominate and cause significant distress or impairment in important areas of functioning but do not fully meet the criteria for any of the dissociative disorders. It has several varieties, one of which is dissociative trance, which we will discuss in our case report, while unspecified dissociative disorders (UDD) refer to symptoms of a dissociative illness that produce severe impairment in critical areas of functioning but lack adequate evidence for a particular diagnosis [1].

Dissociative trance, an example of other specified dissociative disorders (OSDD), is distinguished by a sudden decrease or complete loss of awareness of one's surroundings, leading to excessive unresponsiveness or insensitivity to external stimuli. Minor stereotyped actions (for example, finger gestures) in which the individual is unconscious or unable to control may accompany the unresponsiveness, as well as brief paralysis or loss of consciousness [1]. The patient may express the feeling that his body is moving alone and he has no control over it, even while he is aware of the activity; he might describe it as automation, being controlled by someone else, or even being possessed by some superpower.

In severe circumstances, dissociative trance can result in catastrophic consequences, such as harm to oneself or others. Our case is a tragic illustration of how dissociative trance could occur and the disastrous effects it can have on a person's life. This case report will go into the specifics of the patient's life, the terrible events that precipitated her dissociative periods, and the catastrophic consequences of her psychiatric mental health condition. By investigating our case, we want to bring attention to the seriousness of dissociative trance and the need to get competent aid in psychiatric situations.

## Case presentation

The patient is a 22-year-old married woman; her husband is a second-degree relative. They have three children, the youngest being a newborn of 25 days. The patient didn't have any previous psychiatric disorders, including depression, psychosis, or posttraumatic stress disorder (PTSD). During her early childhood, the patient experienced significant emotional neglect and abuse. Her father was described by the patient as supportive and very affectionate; he cared too much about her and her needs, but he didn’t spend too much time with her because he worked in another city.

She suffered emotional and sexual abuse from relatives, and despite telling her mother about it, the mother did nothing but blame her, which had a negative impact on her trust and personality. She stated that there was no one in her life she could trust and talk to freely during these difficult times. She dropped out of secondary school due to her parents' lack of encouragement to pursue higher education. All of the sexual, emotional, and physical abuse issues she experienced, as well as her difficult childhood and unsupportive family, had a negative impact on her personality. At the age of 16, she accepted being married to a man who was 16 years older than her because she wanted to start a new life away from the hard and poor experiences with her family. After the marriage, the husband neglected and mistreated her; she had been sexually and physically abused by her husband numerous times. Two years after the marriage, the patient was diagnosed with a benign breast tumor, and she was alone during the treatment process because none of her family or husband would support or help her, she reported that none of her family nor her husband knew about her disease. The patient recently initiated a romantic relationship with another man; however, this relationship became complicated when she discovered that her sister had also been in a relationship with the same man, which led to significant emotional distress for the patient.

The patient had three children, a five-year-old boy, a three-year-old girl, and a 25-day-old newborn. Two weeks ago, the newborn died unexpectedly with no clear cause of death. After the death of her newborn, she was very sad, and in a low mood, and she missed him too much despite the fact that the pregnancy was accidental and she didn't want him. In the same week, her husband discovered that she was cheating on him, which caused a conflict leading to increased family disintegration. The patient experienced a significant accumulation of stressors within a short period, including infidelity, unwanted pregnancy, and familial estrangement; this emotional burden was further exacerbated by the tragic loss of her infant child, leading to severe psychological distress prior to the onset of the symptoms.

One week after the death of the newborn, the patient reported a period of decreasing consciousness, a change in the identity, and that an external force controlled her to do specific things, the patient said that she had periods of thinking about killing her daughter. Following that, she reported uncontrollable movements of her hands to hand-choke her daughter, which were controlled by a third force outside her; the patient was not fully conscious during the event and was crying and attempting to pull her hands from the daughter's neck, and after the daughter's death, she was very scared and covered the corpse. The patient tried to leave the house to avoid any injury to her other child if the symptoms occurred again. However, neither the husband nor the family helped her, so she stayed. After a few days, a similar attack of decreased consciousness, changes in identity, and uncontrollable hand movements controlled by an external force occurred, resulting in the hand choking of her five-year-old child.

Clinical tests

During the clinical interviews, her voice was very calm and clear when she talked about her childhood, marriage, and family. When she asked about the death of all her three children, she answered that regret won’t make any difference and that she can start a new life if she finds the right person who can treat her well, and she will try to forget the bad experience she faced. The patient denied any visual hallucinations, auditory hallucinations, delusions, disorganized speech or thoughts, high mood (mania), trouble sleeping, apathy, lack of energy, loss of appetite, or social withdrawal. The patient said that this was God's will and that her daughter had a better life than her. 

The Minnesota Multiphasic Personality Inventory (MMPI) test was performed on three scales (paranoia, schizophrenia, and depression), and all these scales reveal a moderate score, which may reflect the hardness of her life, several recent stressful events, and worrying about the future and judicial rulings. Beck's Depression Inventory Test also revealed a severely depressed mood from the previous hard experience.

Thematic Apperception Test (TAT) is performed to learn more about the patient’s emotions, motivations, and personality. The patient was able to tell many stories on each card and talked more about her struggles and feelings throughout her life, from childhood to the last catastrophic event, particularly when she was shown the empty card. After that, the sentence completion test (SCT) was done, and the patient was interested and collaborated through the test. She reported that the test allowed her to express more about her feelings and thoughts, which also reflect the hard life she lives.

Medical evaluation

The patient underwent a comprehensive medical evaluation. The neurological exam and electroencephalogram (EEG) were both unremarkable. Routine laboratory tests, including a pregnancy test, complete blood count, basic metabolic panel (glucose, electrolytes), and liver function tests (alanine aminotransferase (ALT), aspartate aminotransferase (AST), and alkaline phosphatase), were within normal limits.

The patient's medical, surgical, and psychiatric history revealed no significant findings. She reported no history of chronic medical conditions such as epilepsy, hypertension, or diabetes mellitus. Additionally, there was no history of previous psychiatric illnesses, hospitalizations, substance abuse, suicidal ideation, or head trauma. The patient has no history of any crime. We treated the patient with both pharmacotherapy (including second-generation antipsychotics, antidepressants, and benzodiazepines) and cognitive behavioral therapy (CBT). The patient’s symptoms improved, and follow-up is being conducted.

## Discussion

Dissociative trance disorder (DTD) is categorized under "other specified dissociative disorder" in the Diagnostic and Statistical Manual of Mental Disorders Fifth Edition (DSM-5), which emphasizes altered consciousness and identity disruption without an organic cause [4]. It is classified as a rare disorder in Western countries; it is documented in various cultures worldwide, with a particularly high prevalence in areas where trance or possession experiences are socially or religiously significant [5]. A critical review reported only 402 cases of patients with DTD in the world with an equal ratio between males and females [6]. Studies indicate a significant prevalence of dissociative disorders in non-Western populations, with trance and possession forms being particularly frequent in some Asian and African countries, as well as among specific groups in Europe, such as Italian Catholics who report demonic possession experiences during exorcisms [5,7]. Some of the factors attributed to developing this disease include psychological stressors such as death; some people have an inherited tendency to have psychotic illnesses; and this group of people is at higher risk of developing dissociative disorders. Also, previous traumatic events such as sexual abuse during childhood [7]. 

Dissociative trance disorder (DTD) manifests in many forms. A case study of DTD in Maharashtra highlights the necessity of recognizing the co-existence of DTD and underlying dysthymia [8]. In Indonesia, Tigabinanga culture often associates DTD with cultural rituals like the "Nini Pagar," a rain-inducing ceremony where trance is accepted as a spiritual experience but becomes a medical concern when it causes distress or functional impairment [7]. In a case from Italy, patients experiencing possession-type trances exhibited behaviors such as changes in facial expressions, speaking in unfamiliar languages, and displaying intense emotions like anger or fear. This was differentiated from psychosis by the fact that these patients maintained some connection to reality and did not experience persistent delusions outside of trance episodes similar to the episodes of detachment, delusional thoughts, and temporary changes in identity seen in our case [5].

The pathophysiology of dissociative trance disorder (DTD) involves a disruption in the usual integration of consciousness, memory, and identity, often resulting in involuntary trances that are perceived as spiritual possession. Neurobiologically, dissociative symptoms are understood as protective mechanisms that help the individual cope with trauma or distressing events [4]. This can result in altered states of consciousness that disrupt executive function and sensory perception, often with concurrent memory gaps [4]. Research indicates that DTD may share similarities with dissociative identity disorder (DID), as both involve identity disruptions, although DID involves multiple distinct identities, whereas DTD is characterized by a single altered state [5]. The involvement of specific neural pathways, such as the inhibition of hippocampal regions and activation of the frontal cortex, has been associated with dissociative amnesia and trance states as a means of emotional regulation under stress [7].

Dissociative trance disorder (DTD) is diagnosed by obtaining a full history and analyzing symptoms to distinguish it from other dissociative disorders and psychotic illnesses, such as schizophrenia. The DSM-5 defines DTD as an altered condition that disrupts cultural or religious traditions and causes functional impairment [4,7]. The duration of possession trance episodes can vary greatly, occurring randomly or in response to specific stimuli such as religious or spiritual rituals, emotional suffering, or cultural events. These episodes can last anywhere from a few minutes to several hours and may occur repeatedly [8].

Main clinical features of dissociative trance disorder

Altered Consciousness and Identity Disruption

During a DTD episode, individuals may appear to be in a trance-like state, detached from their usual self-awareness. The patient’s personal identity is often replaced by a distinct state, which may be attributed to external forces or spiritual entities in cultural contexts that recognize possession states. In certain cultural settings, such as in Tigabinanga and Indonesia, DTD may be linked with traditional practices, where the affected individual is believed to be possessed or influenced by spirits following specific rituals [7].

Involuntary Episodes

Unlike culturally accepted trance states that are deliberately entered (e.g., during religious ceremonies), DTD episodes are involuntary and often distressing for the individual and those around them. The episodes are unplanned, and the individual may feel a sense of loss of control, which is a core feature that differentiates DTD from ritualistic trance [5]. As in our case, the patient described involuntary episodes of uncontrollable hand movements to hand-choke her children.

Impairment in Response to External Stimuli

During a DTD episode, individuals may not respond to external stimuli, appearing as if they are "zoned out" or completely unaware of their surroundings. In our case, the patient's child cried and tried to push her hands, but there was no response from the mother. They also might exhibit behaviors such as staring blankly, repetitive movements, or unusual postures. In some cases, these episodes include motor behaviors like twitching, laughing, or crying without an apparent cause. In a study involving patients undergoing exorcisms in Italy, those in possession-type trance states displayed behaviors such as muscle rigidity, sudden changes in tone of voice, and facial expressions of anger or fear [5].

Dissociative Amnesia

Amnesia for trance episodes is common; however, a study found only in 20% of the patients [6] with patients often unable to recall their actions or experiences during the trance state. In many cases, family members or witnesses describe the episodes to healthcare providers, as the affected individuals have little to no memory of what occurred. This memory gap can contribute to confusion and distress once the individual returns to a conscious state [4]. In our case, the patient remembers the episodes but has some degree of amnesia for some details.

Emotional and Behavioral Symptoms

Patients with DTD may experience emotional changes following an episode, including feelings of depression, anxiety as seen in our case, or fatigue. In cases documented in cultural contexts, such as the Tigabinanga case, post-trance symptoms including depressive mood, social withdrawal, and physical fatigue are reported [5].

Cultural Influences on Symptom Expression

Cultural context may play a role in DTD presentations. In some traditions, such as the Nini Pagar ritual in Indonesia, trance states may be interpreted as spiritual experiences. These episodes may involve culturally specific behaviors, like chanting, dancing, or speaking in unknown languages, perceived by the affected individual as communication with spirits or deities [5,7].

Absence of Psychotic Features

Unlike psychotic disorders, individuals with DTD typically do not exhibit persistent delusions or hallucinations outside of the trance episodes. Their trance state may include expressions or behaviors resembling possession or control by an external entity, but they do not usually have the continuous reality distortions seen in schizophrenia or other psychotic disorders [4]. In our case, the patient reported that leaving the house and avoiding contact with her children helped decrease the frequency of the episodes, which is not a typical feature of other psychotic disorders. Table 1 summarizes the differential diagnosis.

**Table 1:** Summary of the differential diagnosis for the case

Aspect	Dissociative Trance Disorder (DTD)	Dissociative Identity Disorder (DID)	Schizophrenia	Postpartum Psychosis
Presence of altered identities	Influence or control by external entities or spirits	Core feature: two or more distinct identities with different behaviors	No alternate identities	No altered identities
Cultural & religious context	Strongly influenced; often linked to possession or spirit beliefs	Not strongly linked to culture	Not strongly linked to culture	Not strongly linked to culture
Temporary or persistent	Temporary; resolves without lasting impairment	Persistent; often lifelong following childhood trauma	Persistent; chronic with episodic exacerbations	Temporary; typically lasts weeks to months postpartum
Clinical features	Decreased or complete loss of awareness of immediate surroundings that presents as profound unresponsiveness or insensitivity to external stimuli	Amnesia, identity fragmentation, dissociation, and mood swings	Delusions, hallucinations, disorganized thinking, flat affect, cognitive deficits	Disorganized behavior, agitation, confusion, insomnia
Hallucinations	NO	NO	Common; auditory hallucinations (e.g., voices) are typical	May include auditory hallucinations (e.g., hearing commands)

Our patient’s clinical presentation met the diagnostic criteria for DTD, and other causes, including psychotic disorders, organic causes, and drugs, were excluded before the diagnosis. Standard care for dissociative trance disorder (DTD) often combines psychotherapy with supportive measures to address underlying psychological distress. Cognitive behavioral therapy (CBT) and grounding techniques are used to help patients regain control during episodes and integrate traumatic memories [7]. Pharmacotherapy may be applied if the patient exhibits comorbid conditions such as anxiety or depression. In Indonesia, case studies have shown that combining psychotherapy with culturally sensitive approaches, such as involving family and traditional healers, can support treatment adherence and efficacy [7].

The prognosis for dissociative trance disorder (DTD) varies depending on the frequency and intensity of trance episodes and the presence of supportive cultural or social structures. Many patients experience reduced episodes over time with treatment, though relapse can occur with stress or exposure to cultural triggers associated with the trance state. Prevention strategies may involve educating communities about the psychological nature of DTD while respecting cultural beliefs. In some cases, such as those involving religious or cultural practices, ensuring that traditional rituals do not lead to prolonged distress or disability may help prevent the transition from culturally accepted trance states to clinical DTD [5,7].

## Conclusions

Dissociative trance disorder (DTD) is a type of other specified dissociative disorder (OSDD) characterized by a sudden decline or complete loss of consciousness. Typically, it involves changes in identity and uncontrollable hand movements described as being controlled by an external force. It is generally associated with amnesia, cultural influences, and the absence of psychotic features; these symptoms occur as protective mechanisms that help the individual cope with trauma or distressing events. In our case, the patient's symptoms led to catastrophic consequences demonstrated by killing two of her children following extreme emotional stress within a short period. The patient was admitted to the hospital and was given second-generation antipsychotics, antidepressants, benzodiazepines, and cognitive behavioral therapy. After close observation, the patient’s symptoms improved, and follow-up is being conducted. This case illustrates that dissociative trance disorder (DTD) can be significant and can lead to catastrophic events, but consequences can be avoided with early identification and appropriate interventions. Further research is needed to provide clear diagnostic criteria and management approaches to prevent such disastrous events.
